# Measuring recognition of body changes over time: A human-computer interaction tool using dynamic morphing and body ownership illusion

**DOI:** 10.1371/journal.pone.0239322

**Published:** 2020-09-18

**Authors:** Myeongul Jung, Jejoong Kim, Kwanguk Kim

**Affiliations:** 1 Department of Computer Science, Hanyang University, Seoul, Republic of Korea; 2 Department of Psychology, Duksung Women’s University, Seoul, Republic of Korea; University of Turin, ITALY

## Abstract

Measuring body image is crucial at both personal and social levels. Previous studies have attempted to quantitatively measure body image but methods for measuring body change recognition over time have not yet been established. The present study proposes a novel human-computer interaction technique using dynamic morphing and body ownership illusion, and we conducted a user study to investigate how body ownership illusion and gender would affect to body change recognition. The results showed that a participant’s body change recognition was weak when the body ownership illusion was strong. In addition, female participants were less sensitive than male participants. With our proposed technique, we demonstrated that we were able to quantitatively measure body change recognition and our empirical data indicated that body change recognition varied depending on body ownership illusion and gender, suggesting that our methodology could not only be used in future body image studies but also in eating disorder treatments.

## Introduction

In several countries and regions, dietary issues are not only affected by physical and/or mental health concerns but are expanding to social and cultural concerns [1]. Previous research have reported that psychiatric symptoms such as anxiety and depression could be caused by frequent exposure to media defining the so called, “ideal” body image [2, 3]. Indeed, numerous people have experienced dissatisfaction with their own bodies, especially their body shapes, simultaneously possessing a strong desire to have an “ideal” body [4]. Thus, various methods have been attempted in several fields, including fashion, nutrition, and psychology, to determine the manner in which people perceive their own bodies. Most studies, however, have focused on the current state of the body and body image, or demonstrated a lack of objectivity. Measuring recognition of body changes over time has not yet been extensively investigated. In this paper, we propose a newly developed human-computer interaction methodology that quantitatively measures body change recognition and describes an empirical study using the new method. Finally, we discuss potential future applications.

### Body image

When we form an image or a picture of our body size and shape in our minds, it is referred to as a body image [5, 6]. A body image is defined in a variety of ways depending on the field of interest. For example, Secord et al. [7] defined it as the satisfaction with each body part and function. Cash [8] proposed a multidimensional self-perception related definition that includes belief, thought, and feeling based on bodily experience. Additionally, Grogan [4] defined body image as a complex concept comprising perceptual (size estimation) and attitudinal (satisfaction, feeling, and behavior) elements.

Several previous studies have attempted to measure body image using a questionnaire [9] and quantitative methods. First, Keizer et al. [10] and Piryankova et al. [11] asked participants to express the size of different parts of their bodies with real or virtual objects. This method is advantageous in that comparison between body parts and objects with real numerical values is possible. However, it is still an indirect measurement through external objects. Second, Thompson et al. [12] had participants choose the closest image to their own body size among several male and female figures of various body sizes. This method is intuitive and easy to conduct. However, participants may focus on the relative comparison between images rather than comparison with their own body size. Furthermore, Grogan [4] argued that female participants may tend to overestimate some specific body parts when they performed tasks using 2-D images, therefore a more sophisticated, computerized technique was proposed. One example using such a computerized technique, had participants choose the body closest in size to their own in a virtual space where various sized 3-D figures were displayed [13]. This methodology has the advantage of being more realistic than 2D figures but is still limited to observing others’ (virtual) figures, not their own. Finally, a recent study [14] scanned the participants bodies to create a virtual avatar. Then a series of different sized 3-D figures were generated from the scanned image (avatar). The participants were then asked to judge whether each avatar looked like themselves. In Mölbert et al.’s [14] method, selecting the body image which has the participant’s own appearance, is more realistic but is still limited in terms of measuring the current state of the body. The various ways for body image measurement mentioned so far have the common limitation that they measure or infer static body images and do not measure the ability to detect body changes.

Body image measurement still provides ample information with implications for the apparel industry or the field of mental health (e.g. eating disorders). For example, the degree of discrepancy between one’s actual body and body image can be assessed. Additionally, it is known that man and woman have different attitudes toward body image. Woman prefer a thinner body while man prefer more a muscular body [4]. Clinical studies [15] have reported that the incidence of eating disorders including anorexia and bulimia nervosa in woman is 10 times higher than in man. Eating disorders tendencies are thought to be correlated with a weakened ability to cope with negative feelings about one’s body (body image flexibility) [16, 17] and in another recent study [18] reported that woman are less flexible regarding negative body image than man. Thus, this gender difference in body image flexibility could be related to the differences in eating disorder incidence between the genders. To prove this possibility, it is necessary to measure the ability to recognize body changes over time, in addition to gathering information about body image at any one time point. In the next section, we review the techniques and phenomena that provided us the theoretical and empirical basis for developing the proposed new human-computer interaction methodology for measuring body change recognition.

### Dynamic morphing technique and body ownership illusion

To develop our methodology, first we adopted a dynamic morphing technique that was established to measure the ability of detecting visual changes in general. The dynamic morphing technique is based on computer graphics and measures one’s sensitivity to changes while a visual object is gradually changing its state from A to B. This methodology has been used in emotional sensitivity studies [19–21]. For example, it is possible to measure the sensitivity to emotional differences by slowly changing a face from a “neutral” to a “happy” face. This sensitivity measurement was also used in a virtual avatar study in which emotional processing in autism spectrum disorder was investigated through the changing emotional state of a virtual avatar [22]. Recently, Mölbert et al. [14] measured participants’ body image using a morphing technique, however, they did not collect the data of the sensitivities to changes in body image over time. Thus, to our knowledge, there has been no study to date that has applied sensitivity to change to research on body image. In the current study, we proposed a new technique for measuring sensitivity to body changes by presenting a virtual avatar with a slowly changing body size.

A virtual avatar is extremely useful in terms of easy manipulation and control by the researcher in body image studies, which is impossible in a real scenario. Additionally, it is possible to make participants feel as though the body of the virtual avatar is their own if the body ownership illusion (BOI) principle is applied to the avatar [23]. The BOI is a phenomenon where one mistakes body parts or objects that do not belong to oneself as one’s own [24–28]. For example, Botvinick et al. [24] reported that observers could mistake an artificial body part (e.g. a rubber-made hand) for their own. Later studies revealed that BOI could be extended more broadly, such as to virtual arms [25] and even to the full-body [26, 27]. The aforementioned studies also found that BOI could induce changes in emotion [26], behavior [27], and even in thought [28].

Recent studies showed that various attributes of one’s body image could be affected by BOI using a virtual avatar, without actual body changes. For example, BOI influenced attitudes toward one’s body [10, 29] and the perception of external object size [30, 31]. Additionally, body image seems to be changed in concert with the body size of the virtual avatar with which BOI is formed [28]. Lastly, a recent clinical study [32] suggested that BOI could be applied to relieve body image distortion in patients with eating disorders. Thus, sophisticatedly generated BOI with a virtual avatar, for example, would provide a practical tool for remediating body image distortion.

In this paper, we propose a new methodology of “virtual reality body change perception measurement” which measures the ability to recognize body size changes in a virtual space, using a dynamic morphing technique and BOI with a virtual avatar. Additionally, we describe an empirical user study to investigate how the new technique worked in an experimental setting. Lastly, we discuss the results from the user study and possible future applications of our new methods, as well as prospective new human-computer interaction techniques.

## Methodology

**Fig 1:**
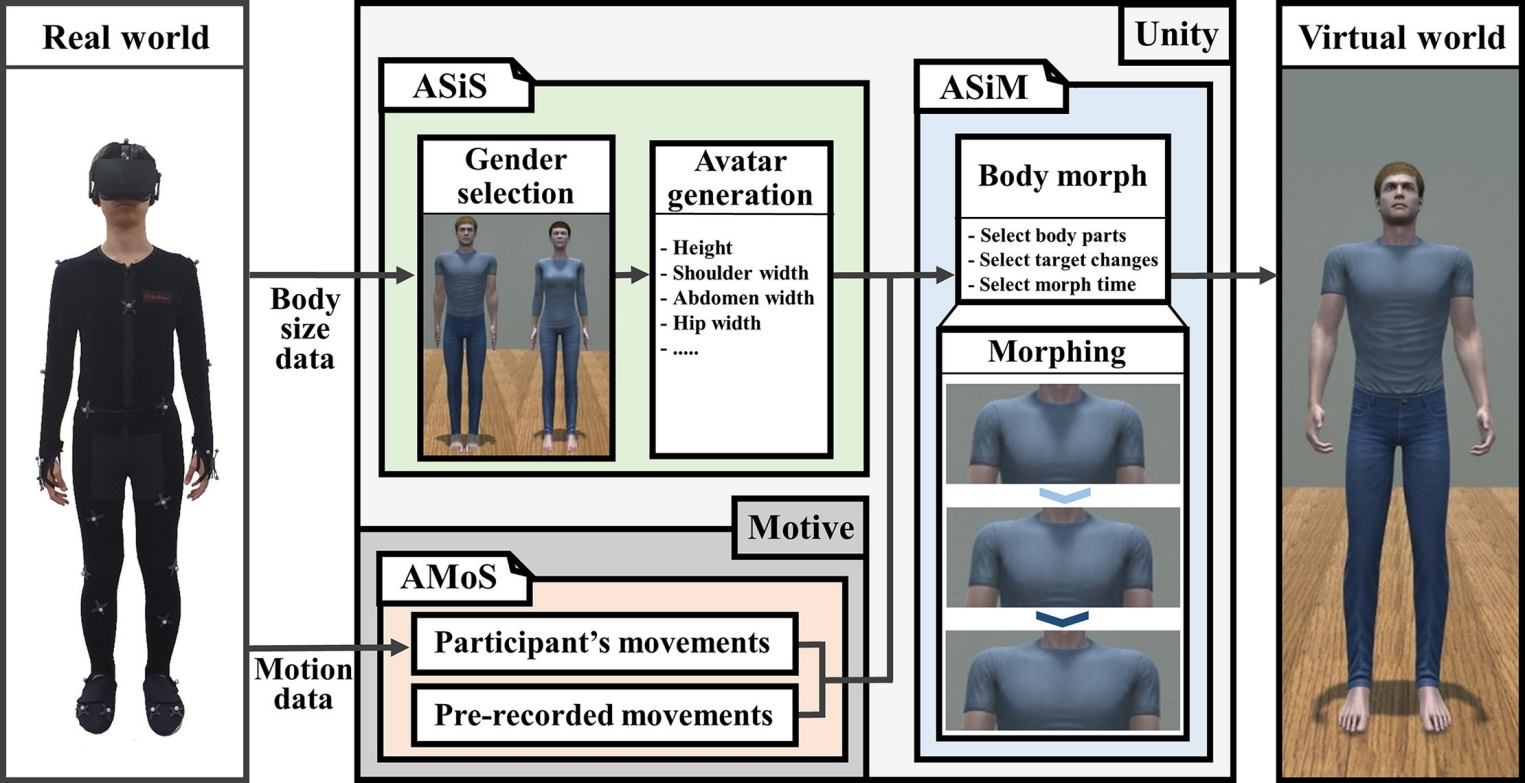
Methodology including avatar size synchronizer (ASiS), avatar size morpher (ASiM), and avatar motion synchronizer (AMoS). The ASiS is designed to make changes to the body size of the virtual avatar using input values from the participant’s actual body size and gender, the ASiM is designed to change the size of the virtual avatar from A to B during a designated amount of time, and the AMoS is designed to adjust the level of motion synchrony.

The methodology that we developed in this study (Fig 1) is based on the Unity 2017.3.1f1 (Unity Technologies, California) and consists of three modules: avatar size synchronizer (ASiS), avatar size morpher (ASiM), and avatar motion synchronizer (AMoS).

**Fig 2:**
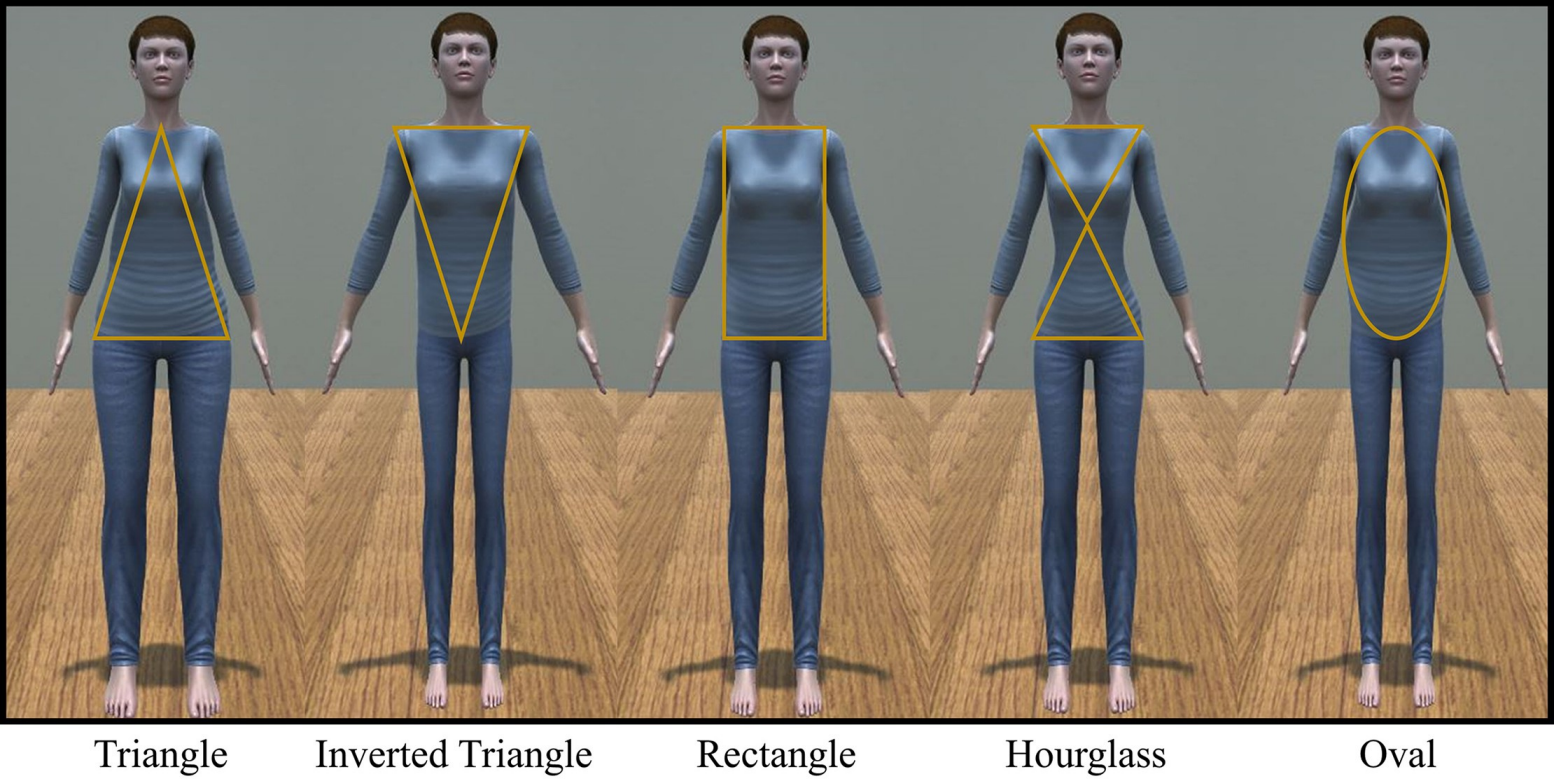
Examples of female avatars with different body sizes.

The first module, ASiS, was designed to make changes to the body size of the virtual avatar using input values from the participant’s actual body size and gender. Body size matching needed to be controlled precisely because mismatches between virtual and real body sizes could alter body perception and induce negative feelings or discomfort [33]. Our ASiS module generated a virtual avatar with similar body size to that of the real body using the height, shoulder, abdomen, and hip widths. Specifically, the size of the entire virtual avatar was rescaled according to the height of the participant and then the shoulder, abdomen, and hip bone widths were additionally rescaled in proportion to the body part values. Fig 2 shows an example of generating female virtual avatars with 5 different body sizes using the ASiS module.

**Fig 3:**
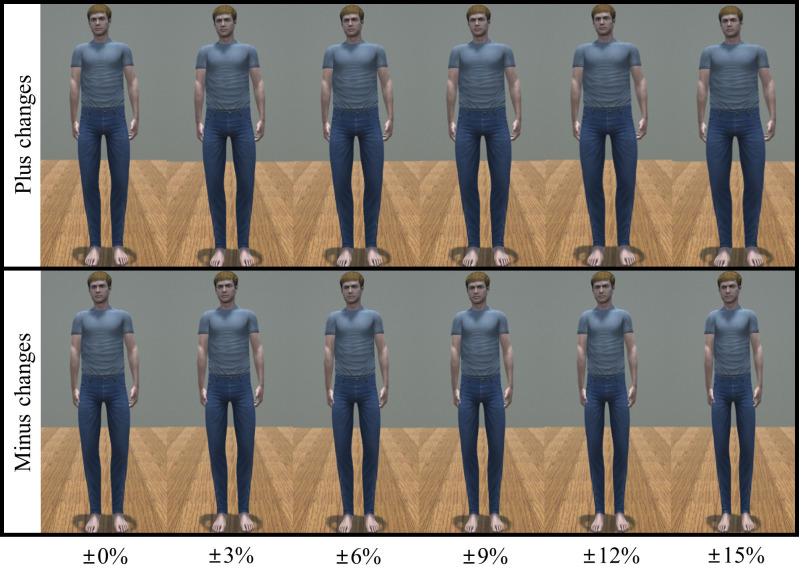
Examples of size morphing in male avatar.

The second module, ASiM, was designed to change the size of the virtual avatar from the participant to program’s target sizes. This is similar to the face morphing method used in [19–21]. Specifically, during a designated amount of time, the size of 18 avatar body parts such as hip, abdomen, chest, shoulder, neck, head, upper and lower legs, feet, upper and lower arms, and hands was gradually changed without changing the height. For example, in our user study, the virtual avatar’s body size changed within a range of 85–115% of the real body size, starting at 100% for 30 seconds. Fig 3 shows an example of morphing the male avatar using ASiM module.

The third module, AMoS, was designed to adjust the level of BOI. We controlled the level of BOI by regulating degree of motion synchrony as suggested in previous studies [26, 27]. In this study, we included synchrony and asynchrony conditions. The synchrony condition used the participant’s movements and the asynchrony condition used predefined movements. Both sets of motion data consisted of 21 bones with six-degrees-of-freedom (6-DOF) for each bone. The motion source was streamed using socket communication to the Unity system at 120 frames per second (fps).

We presented gender-matched avatars in the user study (male avatars to male participants and female avatars to female participants) since the ideal body image could be different between genders [4]. The space for the virtual reality experience was a room of 6 m × 6 m wide. In the virtual space, a 1.5 m (width) × 2 m (height) virtual mirror was in front of the observer (participant) and there were various objects such as a desk, chair, and computer on both sides of the mirror so that the observer could perceive the relative sizes of the objects in the virtual environment [26].

To capture the observer’s movement, we used a motion capture system (Motive 2.0.1, Natural Point, Oregon, USA) and fourteen Flex 13 cameras (Natural Point, Oregon, USA). The Flex 13 cameras were positioned to track the participant’s movement within a space of 4 m (width) × 2.5 m (length) × 2.5 m (height). Each Flex 13 camera had a 1280×1024 pixel resolution, a 120 frames per second frame rate, and a horizontal field of view (FOV) of 56°. During the experiment the participants wore a full-body mocap (motion capture) suit (Natural Point, Oregon, USA) with 37 reflective markers. The positions of the markers within the tracking area were mapped onto the pre-defined baseline skeleton through Motive software and then transformed into motion data. The participants also wore a head-mounted display (Oculus Rift CV1, Oculus, California, USA) which has a resolution of 1080×1200 pixels per eye, a refresh rate of 90Hz, and a diagonal FOV of 110°. Lastly, the software was run on a desktop PC (Windows 10 OS) with Intel® Core™ i7-6700 (Intel, California) CPU, 16GB RAM (Samsung, Seoul), NVIDIA GeForce GTX 1060 6GB (Nvidia, California) GPU.

## User study

To test our new methodology, we conducted a user study on the quantitative measurement of body change recognition. Through the study, we aimed to observe the effect of the level of BOI and gender, which would affect body size recognition over time. We hypothesized that the body change recognition patterns over time will appear differently according to the BOI level and gender.

### Material & methods

#### Ethics statement and participants

The experiment was approved by research site university Institutional Review Board (IRB), and all participants were provided a detailed description of procedure and gave a written consent. The individual in this manuscript Fig 1 has given written informed consent (as outlined in PLOS consent form) to publish these case details. A total of 42 participants (22 female and 20 male participants) were recruited in this user study. The mean (SD) age (years) of the participants was 23.69 (2.19) and all volunteers underwent Symptom Checklist-90-Revised (SCL-90-R) [34].

#### Experiment design

The experiment comprised a 2 × 2 design in which avatar movement synchrony was the within factor (synchrony; sync vs. async) and gender was the between group factor (gender; male vs. female participants). The maximum body size change of the virtual avatar was set to 15% from the starting point (100%) either negatively (thinning) or positively (fattening) [35]. Fig 3 shows an example of virtual avatars with changing body sizes.

#### Experiment procedure

All the participants were given a brief description of the experiment and then completed the participation agreement, pre-questionnaire for demographic information, and SCL-90-R. After the pre-questionnaire was completed, the participants watched a short video clip demonstrating simple gymnastic moves so that they understood what to do in the virtual space. Next, the researcher measured the body size (height, shoulder width, abdomen width, and hip width) followed by the participants’ donning the full body mocap suit.

A 5-min practice session to learn how to move and to perform the tasks in the virtual space occurred before the main session. After taking a break to prevent simulator sickness, the main session began with the virtual avatar that corresponded to the participant’s gender. The main session consisted of two blocks, one block was for the “sync” condition in which the avatar’s movements were synchronized with the participant’s movements and the other block was for the “async” condition. The order of the two blocks was counterbalanced across the participants. Each block had six trials, the initial body size (100%) decreased down to 85% in three trials and increased up to 115% in the other three trials. The six trials occurred in random order. Each trial had two parts. In habituation, BOI was formed (or not) while the participants were asked to move freely and to look at their movements through the mirror. In morphing, body change recognition was measured while participants observed the avatar’s size change for 30 seconds. The participants were asked to press one of the pre-assigned buttons for thinning or fattening, as soon as they recognized the change in avatar size. The participants could change their response without limitation and the perceived body change percentage and direction were recorded. After completing each block, the participants completed the Ownership Illusion Questionnaire (OIQ) and rested for 5 min. After the whole experiment, each participant had a debriefing session. The entire procedure lasted approximately 40 min.

#### Dependent measures

*Body change*. The body change was measured using two subscales. The first subscale involved measuring sensitivity to body changes by presenting a virtual avatar with slowly changing body size. In the current study, it was an average percentage of 6 trials with thinning or fattening avatars, where the participants recognized changes in the body of the virtual avatar. The second subscale was accuracy, which was based on the accuracy ratio (number of correct trials / 6 trials of the current study). If the participant failed to answer correctly (e.g. responding “thinning” while the avatar was “fattening”), it was assumed that the body change was not recognized even at the maximum change (15%). Thus, the maximum change value (15%) was recorded for such trials.

*Ownership Illusion Questionnaire*. We administered OIQ questionnaire [27] after each block to confirm whether BOI was successfully generated with the virtual avatar. The OIQ had two questions on body ownership and one question on agency (refer to Table 1). Each question was a 7-point Likert scale scored from -3 (disagree) to +3 (agree).

**Table 1:** Ownership illusion questionnaire statements.

Variable name	Questionnaire statements	
	LEAST AGREE -3–2–1 0 +1 +2 +3 MOST AGREE	
Ownership	1	I felt that the virtual body I saw when looking down at myself was my own body
Ownership	2	I felt that the virtual body I saw when looking at myself in the mirror was my own body
Agency	3	I felt that the movements of the virtual body were caused by my own movements

#### Data analysis

All data were analyzed using SPSS 25.0. The evaluations of normality were performed using the skewness, kurtosis, and Kolmogorov-Smirnov tests. A 2 (sync vs. async, within factors) × 2 (gender, between factors) repeated measures analysis of variance (ANOVA) was conducted to examine the effects of motion synchrony and gender. If dependent measures did not meet normal distribution criteria, we used a nonparametric estimation. The level of statistically significant *p*-value was set to 0.05.

### Results

Table 2 presents details on the participants’ age and body sizes. The body size measurement in the case of the male participants were all greater than in the case of the female participants (height, shoulder width, abdomen width, and hip width; all *ps* < .001).

**Table 2:** Participants’ age and body sizes analysis.

	Woman (n = 22)	Man (n = 20)	
	M (SD)	M (SD)	*p*
**Age (years)**	23.41 (2.20)	24.00 (2.20)	.389
**Height (cm)**	160.73 (3.24)	176.10 (5.43)	< .001
**Shoulder width (cm)**	35.55 (1.60)	42.15 (1.50)	< .001
**Abdomen width (cm)**	25.73 (1.72)	30.00 (2.18)	< .001
**Hip width (cm)**	29.45 (1.63)	32.00 (1.51)	< .001

M: mean. SD: standard deviation.

#### Analysis of the body change

Table 3 shows the results from the analysis of body changes. Synchrony had the most effect on body change sensitivity. The participants needed more body change for recognition during sync conditions compared with async conditions (*F* (1,40) = 6.610, *p* < .05, *η*^*2*^ = .142; sync mean: 8.22%, async mean: 7.42%).

**Table 3:** Results from analysis of the body changes.

		Female (n = 22)		Male (n = 20)	
		M	SD	M	SD
Sensitivity (%)	Sync	9.29	2.39	7.14	2.49
	Async	8.32	2.19	6.52	1.49
Accuracy (%)	Sync	87.88	18.67	96.67	8.72
	Async	90.91	16.04	93.33	12.57

M: mean; SD: standard deviation; Participants needed a greater amount of body change (sensitivity) for recognition during sync conditions as compared with async conditions (*p* < .05). Furthermore, woman participants were less sensitive to body changes as compared to man participants (*p* < .005). Accuracy results indicated no significant differences in the synchrony conditions and gender (all *ps* > .179).

Additionally, female participants were less sensitive to body change (i.e. needed more change for recognition) than male participants, showing a significant gender effect (*F* (1,40) = 10.864, *p* < .005, *η*^*2*^ = .214; female mean: 8.81%, male mean: 6.83%). However, significant interaction between synchrony and gender was not observed (*F* (1, 40) = .334, *p* = .567, *η*^*2*^ = .008). Body change accuracy analysis with Greenhouse-Geisser correction indicated no significant difference between sync and async conditions (*F* (1,40) = .006, *p* = .938, *η*^*2*^ = .000; sync mean: 92.27%, async mean: 92.12%) and a gender effect was not observed (*F* (1,40) = 1.875, *p* = .179, *η*^*2*^ = .045; female mean: 89.39%, male mean: 95.00%). Lastly, interaction between synchrony and gender was not observed (*F* (1, 40) = 2.718, *p* = .107, *η*^*2*^ = .064).

#### Analysis of OIQ

Table 4 shows the results from the OIQ score analysis with Greenhouse-Geisser correction. For the two body ownership questions, the participants reported a stronger body ownership experience during the sync condition compared with the async condition (OIQ 1: *F* (1, 40) = 67.332, *p* < .001, *η*^*2*^ = .627; OIQ 2: *F* (1, 40) = 37.744, *p* < .001, *η*^*2*^ = .485). However, the gender effect was not observed in the answers to these questions (OIQ 1: *F* (1, 40) = 2.393, *p* = .130, *η*^*2*^ = .056; OIQ 2: *F* (1, 40) = 1.885, *p* = .177, *η*^*2*^ = .045). For the question on agency, the participants reported strong feelings of ownership to the avatar’s movements during the sync condition as opposed to the async condition (OIQ 3: *F* (1, 40) = 98.314, *p* < .001, *η*^*2*^ = .711). Differences between the genders was not observed for this question (OIQ 3: *F* (1, 40) = 1.776, *p* = .190, *η*^*2*^ = .043). Across all the three questions, significant interaction between synchrony and gender was not observed (OIQ 1: *F* (1, 40) = 1.594, *p* = .214, *η*^*2*^ = .038; OIQ 2: *F* (1, 40) = .001, *p* = .974, *η*^*2*^ = .000; OIQ 3: *F* (1, 40) = .068, *p* = .796, *η*^*2*^ = .002).

**Table 4:** Results from the ownership illusion questionnaire analysis.

		Female (n = 22)		Male (n = 20)	
		M	SD	M	SD
OIQ1	Sync	1.41	.96	1.65	.99
	Async	-1.05	1.81	- .15	1.81
OIQ2	Sync	.91	1.11	1.40	1.19
	Async	- .77	1.63	- .30	1.75
OIQ3	Sync	2.18	.66	2.55	.61
	Async	-1.46	2.11	- .90	2.27

M: mean; SD: standard deviation; OIQ: Ownership Illusion Questionnaire; Participants reported stronger body ownership experiences during synch condition as compared to async conditions (all *ps* < .001); however, no gender effect was observed in these questions (all *ps* >.130).

We have quantitatively measured body change recognition using the proposed methodology and investigated how the BOI level and gender affects the recognition of body changes. Overall, the participants could recognize body size changes when the avatar’s body either increased or decreased by 7.86% changes (The overall average value for body change recognition was calculated by collapsing all the sensitivity values of the conditions and participants.). In addition, participants were less sensitive to body changes during sync (stronger BOI) than async (weaker BOI) conditions and female participants were less sensitive than males, supporting our hypothesis. Our results further suggest that the factors affecting body image found in previous studies [28, 29] also affect perception of body size changes. Thus, our methodology could be widely applied to future body size change recognition studies as well as body image studies.

## General discussion

In the present study, we proposed a new methodology for measuring body size change recognition using a dynamically morphing avatar and BOI. We conducted a user study to confirm the usefulness of the new technique.

The first finding from the user study was that the BOI level affected body size change recognition differently. Specifically, recognition sensitivity became weakened during the sync condition regardless of gender. It is thought that higher BOI levels facilitate response to visual and auditory stimuli, as reported by several previous studies [36–38]. In this context, our results may seem contradictory to the previous findings, however, it is necessary to consider the role of proprioception associated with strong BOI [39]. The participants during sync conditions seem to have somewhat overlooked body changes compared to during async conditions, while unconsciously updating proprioception at the same time. This eventually resulted in more stable maintenance of proprioception over time. Previous studies on BOI and static body image suggested a similar idea to the current results. For example, van der Hoort [30] and Banakou [31] found that the formation of stronger BOI induced rescaling phenomena in size perception depending on the body size of virtual avatar. Other studies showed that desired body images can be modified through higher BOI levels [28, 29, 31, 40]. Riva [23] suggested a predictive coding [41], which is a brain mechanism that minimizes the discrepancies between sensory input and internal representation, to explain the aforementioned phenomena. In other words, the participants may have unconsciously adjusted the perception of their own bodies to regulate and control their bodies more efficiently in the virtual environment using the predictive coding mechanism. Taken together, our results suggested that people with a higher BOI levels could become less sensitive to body size changes over time. By unconsciously adopting predictive coding, they achieve a more stable perception, even though higher BOI levels usually facilitate response to visual stimuli. In contrast, the predictive coding mechanism may not be important during async conditions (lower BOI). A future application of this work could be developed to treat eating disorders, with special attention to async conditions. Under async conditions people formed a low level of BOI with the avatar and showed greater sensitivity to body size changes. Therefore, application of this condition could make people with eating disorders more sensitive to their positive as well as negative body changes, which could provide motivation for changing eating habits. However, we also need to consider another possibility that synchronicity with the virtual avatar can independently affect both BOI and change recognition. Movement involves an increase in attention and can independently distract a participant’s ability to notice the body changes in the avatar. We believe that this possibility should be considered in further research.

The second major finding from the user study was the different recognition sensitivities to body changes between male and female participants. Female participants required approximately 2% more change in body size for successful recognition compared with male participants. One may think that this result seems to be counterintuitive because it is known that women show greater interest in appearance [42] and have lower appearance satisfaction [43] in general. We speculate that the difference in sensitivities between the genders could be related to body image flexibility, which is the ability to cope with negative thoughts, feelings, and sensations toward the body [18]. Males are generally more satisfied with their appearance. This enables them to readily perceive and respond more flexibly to both positive and negative body changes. On the contrary, as women have both low body image flexibility and low appearance satisfaction, they face difficulty coping with negative thoughts, resulting in low sensitivity to body changes or unconscious to the avoidance of the perception of these body changes. Indeed, it was previously reported that women show lower body image flexibility than men [18]. Our body change sensitivity has similar tendencies to that of body image flexibility. This also provides some speculation on why women are more vulnerable to eating disorders and are less likely to be treated successfully. If a person who has low body image flexibility and satisfaction is in a high-risk state of eating disorder with ongoing body changes, this person may be more vulnerable because they have a lower sensitivity to further body changes compared with people with a higher body image flexibility. Additionally, if a person in an eating disorder state has low body image flexibility, this person would have difficulty in escaping from the disorder due to the poor perception of positive signals in the body caused by medical or psychological treatments. Therefore, although speculative for now, we suggest that our methodology for measuring body change recognition could be used for developing or improving treatment programs for eating disorders.

It is worth noting the strengths of the new technique we developed in this study. Recent studies have attempted to generate BOI with more advanced, personalized virtual avatars created by 3D scanning the participants [14, 44]. However, because of the high cost and the large amount of time needed to create personalized virtual avatars, many studies still use standardized avatars [10, 26–28, 30–32]. In our study, we also manipulated participant BOI using a standardized avatar. Regardless, one of the strengths of our methodology is the expression of various body size patterns, especially using the ASiS module. This customization using standard avatars would provide a useful alternative to personalizing virtual avatars at a low cost and using less time. Furthermore, although the current ASiS module only makes inexact virtual to real body size matches for now, the module’s functionality can be easily extended by the addition of a 3-D scanner for precise body and face matching. Another feature worth noting is that the ASiM module was used to morph the width of the shoulders, abdomen, and hip in our study. In fact, the ASiM module makes it possible to change up to 18 body parts separately depending upon the experimental purpose. Thus, in future studies, this function of the ASiM module could be used for measuring sensitivity to change in a specific body part. For example, we could investigate whether change recognition would be dependent upon body parts and/or the participant’s gender. Lastly, the AMoS module was used to induce BOI levels by two motion synchrony levels (sync and async). Recently, Choi et al. [45] attempted to use three synchrony levels and showed that there were significant differences in BOI, presence, and simulator sickness among those three levels. A future study measuring body change sensitivity with more levels of BOI would be of interest.

There are limitations in the present study. First, our participants were sampled from young adults without any physical and mental health issues. Follow-up studies should recruit participants of diverse age, race, and mental status to confirm and generalize the effect of BOI and gender observed in the present study. In addition, a follow-up study with eating disorder patients or high-risk people suffering from body image distortion would provide us further understanding of recognition characteristics of body changes in these people compared with healthy controls. Second, our participants showed significant differences in actual body sizes between gender groups, which may have affected the main effect of gender on body change sensitivity. Future studies should confirm the results from the current study by comparing gender groups without differences in body size. Finally, we found that body change sensitivity can be used as a quantitative measurement for body image flexibility. But, there was no assessment of body image flexibility in the current study. Future studies should confirm the relationship between body change sensitivity and body image flexibility.

## Conclusion

In the present study, we developed a new methodology for quantitatively measuring the recognition ability of body changes over time and conducted a user study to confirm the usefulness of the technique. The results from the user study showed that recognition ability could be regulated by the level of BOI and manifested differently between gender. This also suggested that the observed gender differences in terms of attitude toward body image could be derived from different sensitivities between genders to body changes. Our results provide empirical data for developing or improving cognitive-behavioral therapies that make women more sensitive to positive change, and furthermore, could be applied to a wide range of programs including body image distortion treatment, personal and related social issues.

## Supporting information

S1 Dataset Measuring recognition of body changes over time_data. (XLSX)
